# Prompt gamma timing for proton range verification with TlBr and TlCl as pure Cherenkov emitters

**DOI:** 10.1088/1361-6560/ad4304

**Published:** 2024-05-14

**Authors:** Justin Ellin, Leonor Rebolo, Michael Backfish, Eric Prebys, Gerard Ariño-Estrada

**Affiliations:** 1 Department of Biomedical Engineering, University of California Davis, CA, United States of America; 2 Crocker Nuclear Laboratory, University of California Davis, CA, United States of America; 3 Department of Physics, University of California Davis, CA, United States of America; 4 Institut de Física d’Altes Energies—Barcelona Institute of Science and Technology, Bellaterra, Barcelona, Spain

**Keywords:** prompt gamma timing, TlBr, TlCl, Proton therapy range verification

## Abstract

*Objective*. Prompt gamma timing (PGT) uses the detection time of prompt gammas emitted along the range of protons in proton radiotherapy to verify the position of the Bragg peak (BP). Cherenkov detectors offer the possibility of enhanced signal-to-noise ratio (SNR) due to the inherent physics of Cherenkov emission which enhances detection of high energy prompt gamma rays relative to other induced uncorrelated signals. In this work, the PGT technique was applied to 3 semiconductor material slabs that emit only Cherenkov light for use in a full scale system: a 3 × 3 × 20 mm^3^ TlBr, a 12 × 12 × 12 mm^3^ TlBr, and a 5 × 5 × 5 mm^3^ TlCl. *Approach*. A polymethyl methacrylate (PMMA) target was exposed to a 67.5 MeV, 0.5 nA proton beam and shifted in 3 mm increments at the Crocker nuclear laboratory (CNL) in Davis, CA, USA. A fast plastic scintillator coupled to a photomultiplier tube (PMT) provided the start reference for the proton time of flight. Time of flight (TOF) distributions were generated using this reference and the gamma-ray timestamp in the Cherenkov detector. *Main results*. The SNR of the proton correlated peaks relative to the background was 20, 29, and 30 for each of the three samples, respectively. The upper limit of the position resolutions with the TlCl sample were 2 mm, 3 mm, and 5 mm for 30k, 10k, and 5k detected events, respectively. The time distribution of events with respect to the reference reproduced with clarity the periodicity of the beam, implying a very high SNR of the Cherenkov crystals to detect prompt gammas. Background presence from the neutron-induced continuum, prompt gammas from deuterium, or positron activation were not observed. Material choice and crystal dimensions did not seem to affect significantly the outcome of the results. *Significance*. These results show the high SNR of the pure Cherenkov emitters TlBr and TlCl for the detection of prompt gammas in a proton beam with current of clinical significance and their potential for verifying the proton range. The accuracy in determining shifts of the BP was highly dependent on the number of events acquired, therefore, the performance of these detectors are expected to vary with different beam conditions such as current, pulse repetition, and proton bunch width.

## 1. Introduction

The number of centers offering proton radiotherapy (PR) for cancer is increasing significantly worldwide (Ngwa *et al* 2023). PR poses additional challenges to conventional radiotherapy, namely dose conformity hurdles as a result of uncertainty in the end of proton Bragg peak (BP) (Liu *et al* 2021), which is a major concern as it might expose critical organs nearby the cancerous tissue. Proton range verification (PRV) via prompt gamma imaging (PGI) has been studied through different methods during the last decade (Krimmer *et al* 2018, Aleksandra Wrońska and for the SiFi-CC group 2020). PGI methods based on a mechanical collimation of the prompt gammas have been tested with patients and show PRV accuracies down to 1 mm (Richter *et al* 2016, Xie *et al* 2017, Berthold *et al* 2021). These systems, however, require high volumes of heavy high Z materials to stop most of the prompt gammas that require a large apparatus with very limited positioning flexibility.

Prompt gamma timing (PGT) is another PGI modality that uses the time-of-flight information of the proton and of the prompt gamma to detect position shifts of the Bragg peak (Golnik *et al* 2014). PGT has been evaluated in clinical proton beams of up to 227 MeV with prompt gamma detectors consisting of scintillation crystals using the radiofrequency (RF) of the accelerator as the reference signal (Hueso-González *et al* 2015, 2018). Previous reports show the RF signal shifts over time with respect to the proton bunches and introduces a significant uncertainty (Petzoldt *et al* 2016). For this reason, in most recent publications reference detectors close to the beam nozzle were used instead of the RF signal to improve the PGT measurements with the similar scintillation-based detectors (Pausch *et al* 2016, Werner *et al* 2019).

Alternatively to scintillation crystals, PbF_2_, a pure Cherenkov emitter, has been studied for the first time for PGT very recently (Jacquet *et al* 2024). These measurements were acquired at single proton regime (SPR), where the beam is tuned to deliver on average a single proton per bunch. In that setting, the signal induced by the proton in the T_0_ and gamma timestamp can be unambiguously matched such that each event conveys information about BP positioning independently. With this approach, a 4 mm range uncertainty (2*σ*) was obtained with a dataset of 600 prompt gamma detections in a cyclotron with 63 MeV protons.

In this work we report on PGT measurements using two pure Cherenkov emitter materials, thallium bromide (TlBr) and thallium chloride (TlCl), in a 67.5 MeV proton beam. Due to their high densities and index of refraction, both crystals generate comparable or greater Cherenkov light yield than PbF_2_ (Rebolo *et al* 2024). TlBr and TlCl are semiconductor materials and thus offer the possibility to collect the signals generated by the electrons to obtain accurate measurements of the energy and interaction point in 3D simultaneous to the detection of the Cherenkov light (Ariño-Estrada *et al* 2019, 2021b). Both the RF of the cyclotron and a start detector were recorded as a reference while the beam was operated at currents of around 0.5 nA. The measurements were performed at the Crocker nuclear laboratory (CNL), in the campus of the University of California, Davis (UCD), where eye cancer proton therapy treatments have been done at CNL at the same energy and beam currents for over 25 years (Daftari *et al* 1996). This article presents the first PGT measurements with pure Cherenkov emitters acquired at a realistic proton beam current and including simultaneously intensity and detection time information.

## 2. Materials and methods

### 2.1. Environment setup at Crocker nuclear laboratory

**Figure 1.**
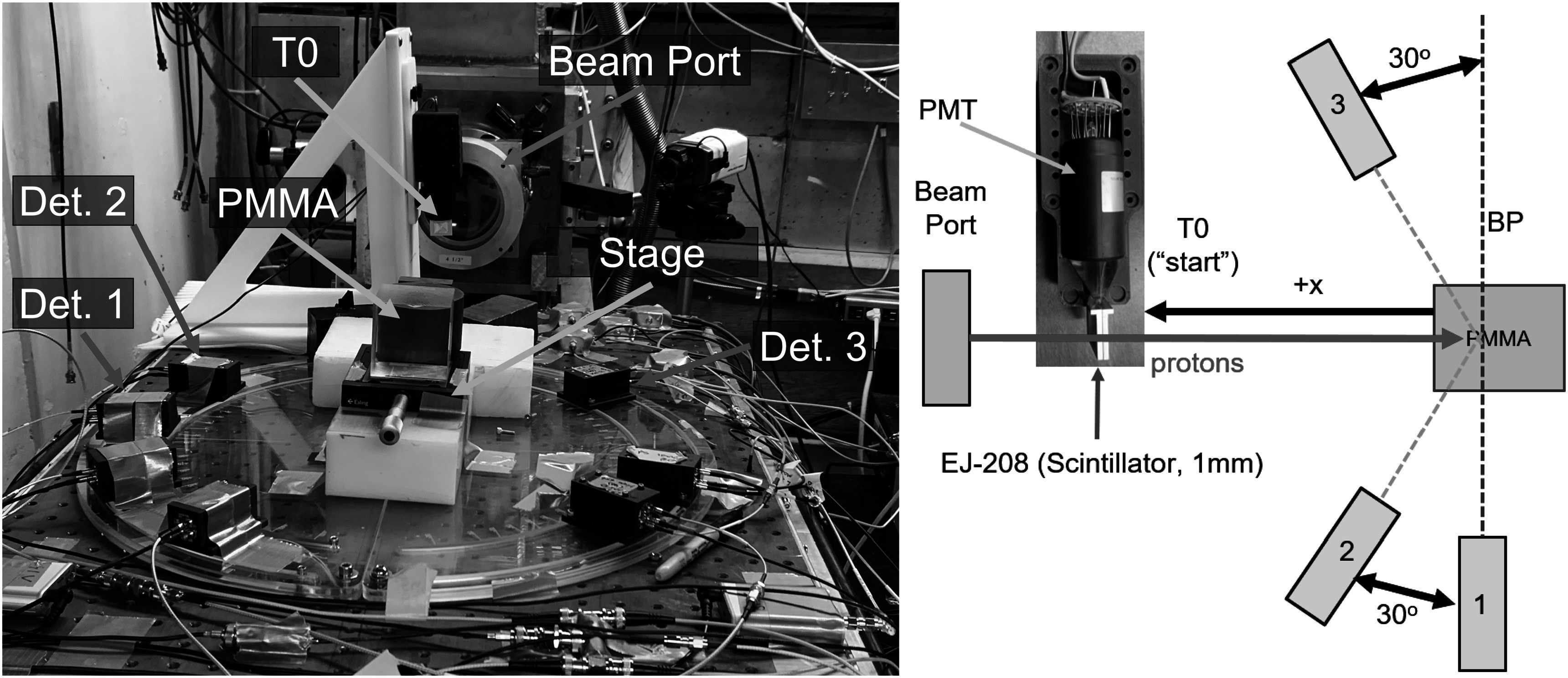
Experimental setup at CNL (left) and schematic representation (right) of T_0_ detector with photomultiplier tube (PMT), PMMA target, and prompt gamma detectors used in this analysis. (1) 3 × 3 × 20 mm^3^ TlBr (‘TlBr20’), (2) 12 × 12 × 12 mm^3^ TlBr (‘TlBr12’), (3) 5 × 5 × 5 mm^3^ TlCl (‘TlCl5’).

Data were acquired at the 76 inch cyclotron at CNL, in the UCD campus. This is a variable energy, variable species cyclotron, which was operated at the maximum proton energy of 67.5 MeV for this experiment, corresponding to a 22.5 MHz bunch frequency. While this energy is lower then the 200–300 MeV used in patient treatments, the proton induced prompt gamma production cross sections in tissue are negligible beyond 40 MeV (Verburg and Seco 2014) and their emission at the end of the proton range is very similar. Figure 1 shows a picture and a schematic illustration of the acquisition setup.

Four detectors were used for this experiment: a reference detector (T_0_ in figure 1), and three prompt gamma detectors (1, 2, and 3 in figure 1). Only three of the detectors in the photograph were used for this study. The radio frequency signal from the cyclotron was also acquired. A polymethyl methacrylate (PMMA) target with dimensions of 5.08 × 5.08 × 5.08 cm^3^ (2 × 2 × 2 in^3^) was used to generate a BP in a tissue equivalent material and moved with a linear stage to vary the proton travel distance and thus, the delay between the start and stop signals. A beam spot size of approximately 1.5 cm (2*σ*) was directed to the center of the face of the PMMA target. The spot size and location were verified through use of a phosphor screen used for beam alignment before measurements were taken. Approximately 5 min of data were acquired for each target position.

### 2.2. Radiation detectors

The start detector (hereafter referred to as ‘T_0_’) consisted of a 10 × 10 × 1 mm^3^ EJ-208 plastic scintillator (Eljen Technology, Sweetwater, TX, USA) coupled to a H10580 photomultiplier tube (PMT) (Hamamatsu Photonics KK (HPK), Hamamatsu, Japan). Two materials were studied as stop, or prompt gammma, detectors: TlBr and TlCl. Both have high density (>7 g cm^−3^) and atomic numbers due to the presence of Tl (*Z *= 81), and therefore also high index of refraction (2.6 and 2.3 at 570 nm, respectively) (Ariño-Estrada *et al* 2021a). Additionally, their cutoff wavelengths (3̃80 nm for TlCl, and 440 nm for TlBr) makes them also suitable Cherenkov emitters for gamma energy depositions of at least a few hundred keV. Earlier measurements using similar TlBr and TlCl crystals, same SiPMs, and comparable readout electronics achieved a coincidence time resolution with 511 keV photons between 300 and 400 ps (Ariño-Estrada *et al* 2021a). These time resolutions are well below the expected 2–3 ns proton bunch width and thus are a good fit for this experiment.

**Figure 2.**
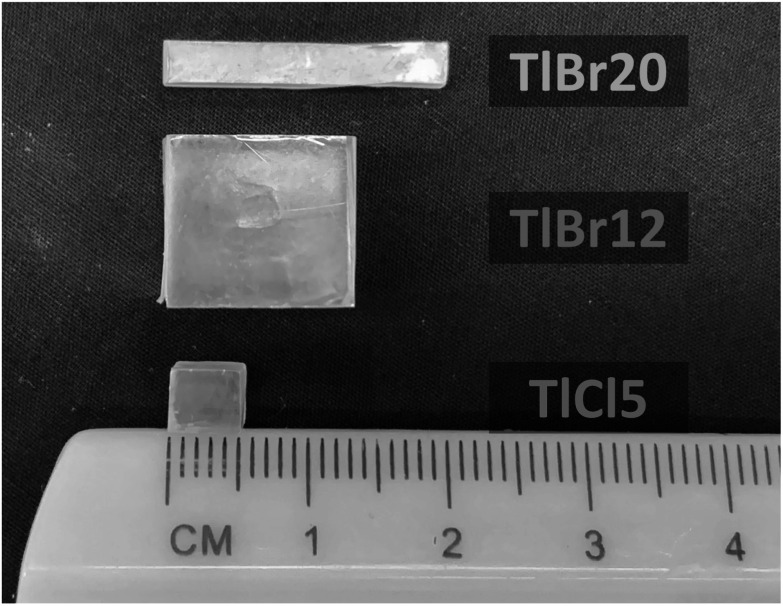
Each of the samples used in this work labeled as defined in figure 1.

Three crystals with different dimensions were used (figure 2): a 3 × 3 × 20 mm^3^ TlBr (referred to as TlBr20), a 12 × 12 × 12 mm^3^ TlBr (referred to as TlBr12), and a 5 × 5 × 5 mm^3^ TlCl (referred to as TlCl5).

All three were placed on fixed positions at approximately 20 cm from the target (figure 1). The smaller TlBr20 and TlCl5 crystals were coupled with 3 × 3 mm^2^ Hamatsu Silicon Photomultipliers (SiPMs) S14160-3050HS while TlBr12 was coupled with a 6 × 6 mm^2^ Hamamatsu SiPM S14160-6050HS to cover more surface. The SiPMs were readout using Broadcom (San Jose, CA, USA) AFBR-S4K evaluation boards. Their output was amplified with ZFL-1000LNB+ RF low noise amplifiers from Mini-Circuits (Brooklyn, NY, USA) before digitization. All SiPMs were biased at 41 V and operated at room temperature.

The RF, T_0_, and gamma-ray waveforms were recorded using two daisy-chained DRS4 evaluation boards from Paul Scherrer Institute (Villigen, Switzerland). The signals of the prompt gamma detectors (TlBr12, TlBr20, and TlCl5) were used as triggers. Their trigger thresholds were set at 100 mV and an OR logic with the three of them was used. For each event 5 waveforms were recorded: the RF signal, the T_0_, and the three prompt gamma detectors. The record length and sampling frequency was 200 ns and 5 GS s^−1^, respectively, for all waveforms.

### 2.3. Analysis

**Figure 3.**
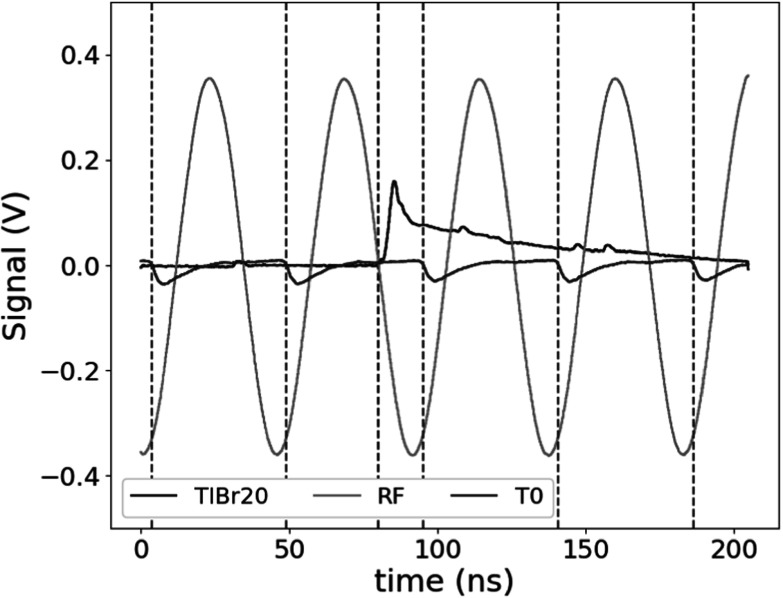
Representative waveform from the TlBr20 crystal (blue), cyclotron RF monitor (gray), and T_0_ (maroon). Gamma timestamp and possible T_0_ reference indicated with dashed lines.

For each prompt gamma detector, 30k events were acquired at each target position. The timestamp for each of the prompt gamma detectors was determined through the use of a leading edge threshold set above the noise of the signal after baseline subtraction. Figure 3 shows a representative event indicated in blue with a prompt rise associated with the Cherenkov emission followed by the decay time of the SiPM and preamplifier. Pileup from this decay was found to be insignificant given the efficiency of the setup.

For the T_0_ detector, each waveform contained between 4 and 5 regular pulses within the trace of each event, each of them corresponding to one proton bunch. The baseline was determined through the use of a linear fit on the tail of the proceeding pulse. The timestamp for each pulse was then again determined through a leading edge threshold after inversion and subtraction of this baseline. These are indicated by maroon dashed lines in figure 3. To choose the best reference, a consistency cut was applied to keep only pulses for which the timestamp was within 1 ns of an integer multiple of the beam period for any other T_0_ pulse. After the cut, the T_0_ pulse with the fastest rise time to 15 mV after crossing the leading edge threshold was selected as the start reference. The proton time of flight was determined as the difference between the T_0_ and gamma detector timestamps modulo the cyclotron beam period.

## 3. Results

### 3.1. SNR study

**Figure 4.**
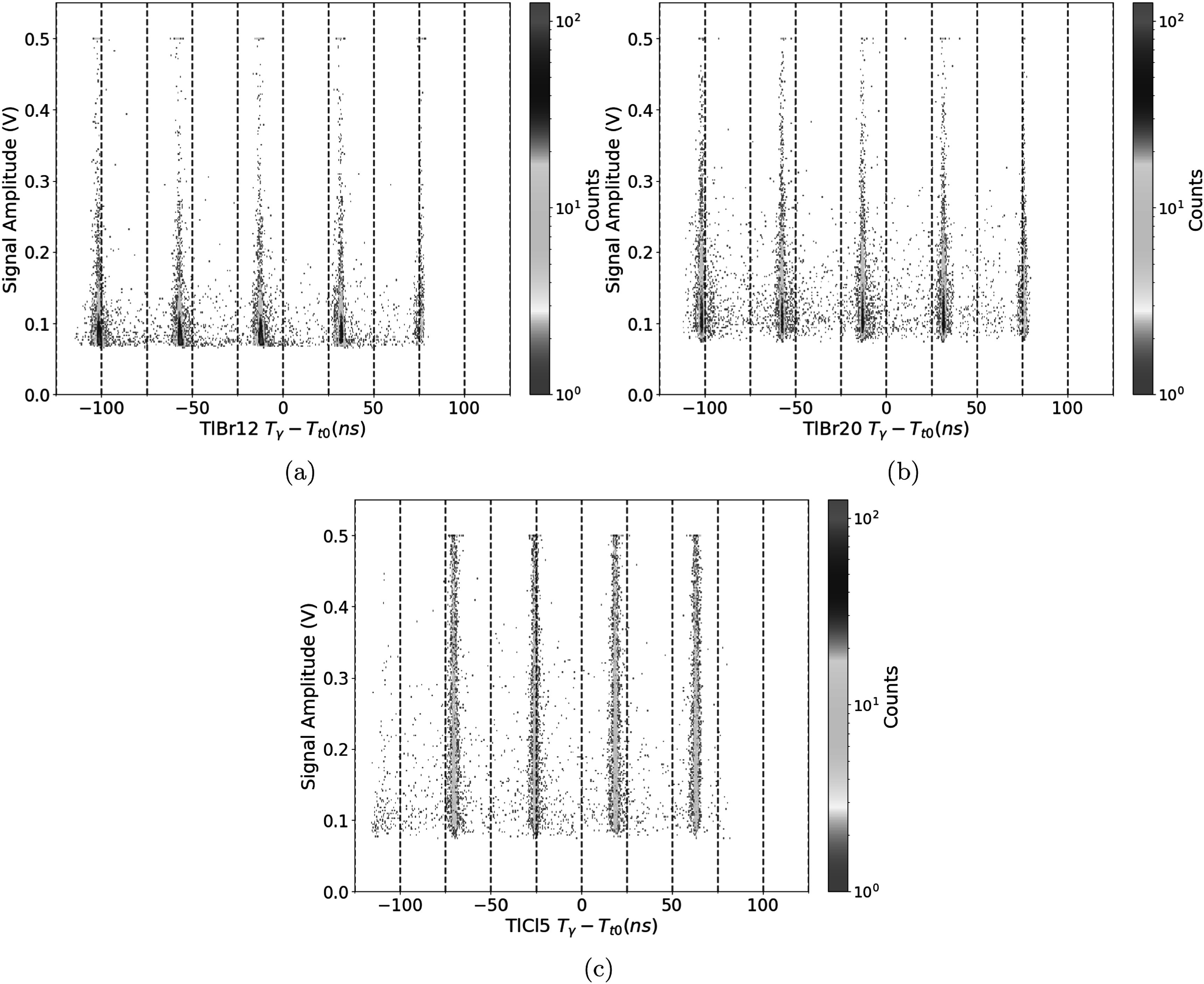
Cherenkov intensity versus timestamp for events detected in (a) 12 × 12 × 12 mm^3^ TlBr crystal, (b) 3 × 3 × 20 mm^3^ TlBr crystal, (c) 5 × 5 × 5 mm^3^ TlCl crystal.

**Figure 5.**
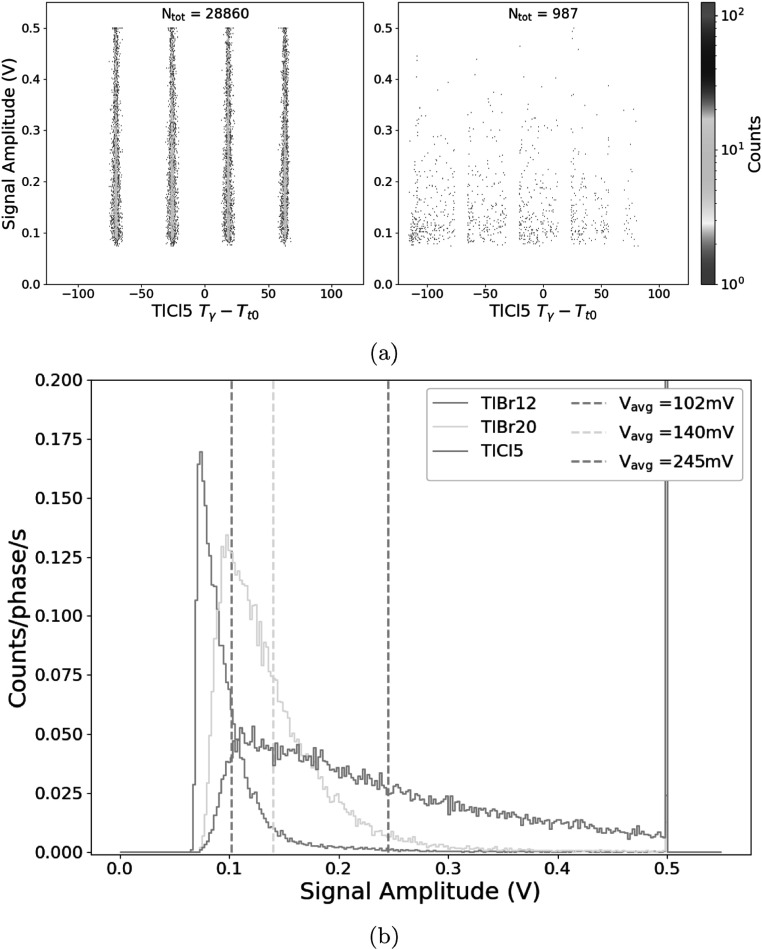
(a) Example selection of signal and background ROIs for 5 × 5 × 5 mm^3^ TlCl. Signal is defined as within 5 ns around the time points with the maximum number of events with background as all remaining events. (b) Signal amplitude projections of the Cherenkov detectors with ROIs of 5 ns centered on each of the prominent time of flight bands in figures 4(a)–(c). The counts were normalized by the total time spanned by all the ROIs and by the total acquisition time.

Figures 4(a)–(c) show an example of the uncorrected time difference between the T_0_ and gamma timestamp for all crystals plotted against the signal amplitude for 30k events over the span of about 5 min. The bands were spaced with the periodicity of the cyclotron RF for all figures. These narrowed at higher photon counts for the TlBr12 and TlBr20, while the event density was more homogeneous along the *Y* axis in the TlCl5 case. One can note there were very few events between each of the bands. These bands were separated by the cyclotron period of 44.4 ns. Regions of interest (ROIs) of 5 nanosecond width centered on each of these peaks were selected as illustrated for TlCl5 in figure 5(a). The peak regions were then summed along the *y*-axis. These projections were normalized by the the total width of the bins and presented in figure 5(b). Signal-to-noise ratio (SNR) was calculated as the number of counts within this ROI over the number of counts outside of this ROI averaged across all three positions and found to be 20.2, 29.2, and 29.5 for the TlBr20, TlBr12, and TlCl5 samples, respectively. These results are summarized in table 1 including the average peak intensity for each of the samples.

**Table 1.** Summary of SNR parameters for each crystal. ${\overline{N}}_{\mathrm{peaks}}$ and ${\overline{N}}_{\mathrm{bkg}.}$ are the number of counts in the peak and background ROIs, respectively, across all positions as seen in the example of figure 5(a). ${V}_{\mathrm{avg}}^{\mathrm{peak}}$ is the average maximum signal intensity for the peak ROI as seen in figure 5(b).

Sample	Acq. time (s)	${\overline{N}}_{\mathrm{peaks}}$	${\overline{N}}_{\mathrm{bkg}.}$	SNR	${V}_{\mathrm{avg}}^{\mathrm{peak}}$ (mV)
TlBr20	267	28 548	1414	20.2	140
TlBr12	487	28 980	991	29.2	102
TlCl5	311	28 875	979	29.5	245

### 3.2. PGT measurements

**Figure 6.**
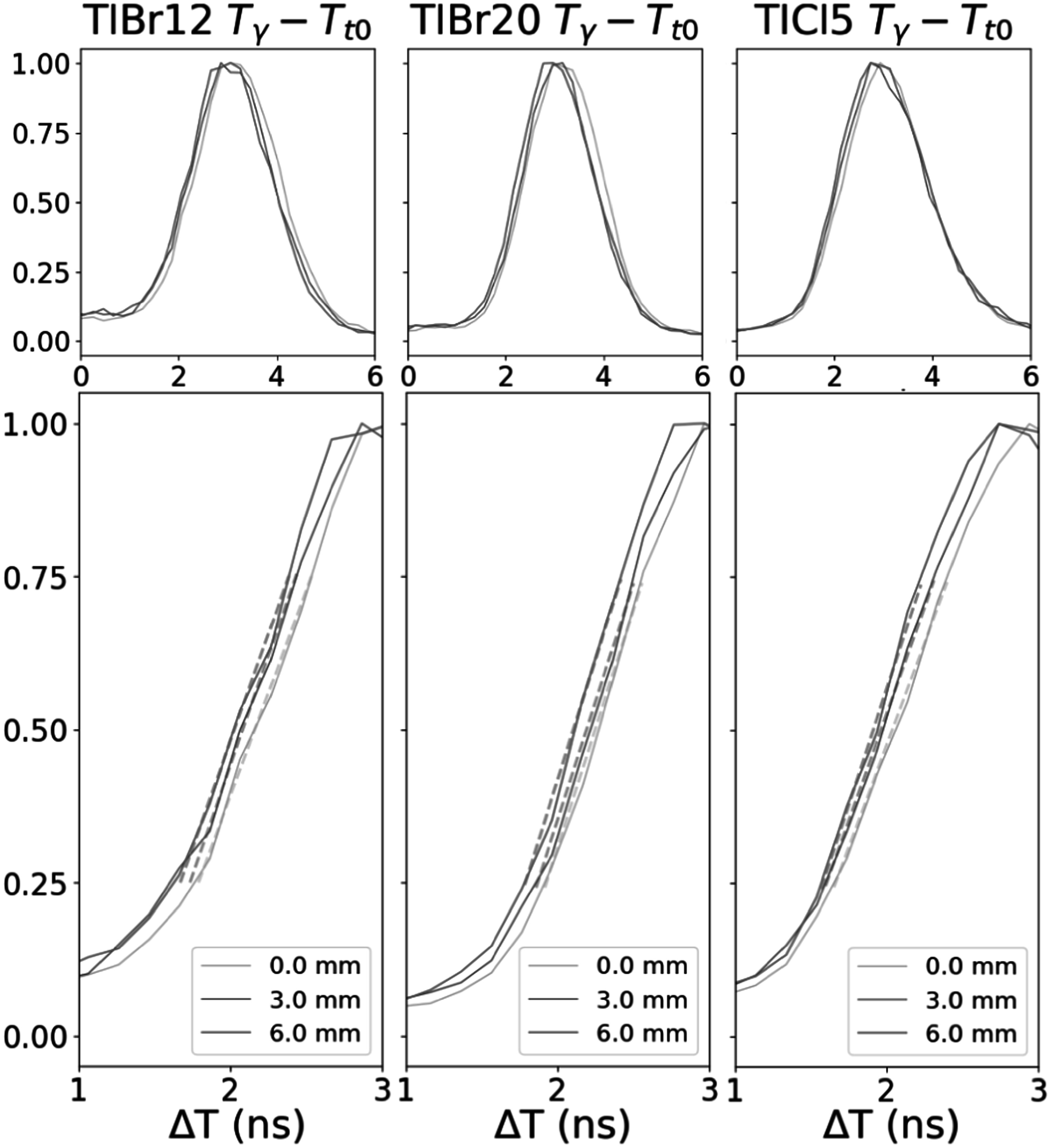
Proton time of flight distributions for each of the crystals at 0 mm, 3 mm, and 6 mm PMMA positions. Each distribution was normalized to the maximum count bin. Top row shows the full distribution for each detector. The bottom row shows a close up of the rising edges for each as well as linear fits on the 25%–75% rising edge.

**Figure 7.**
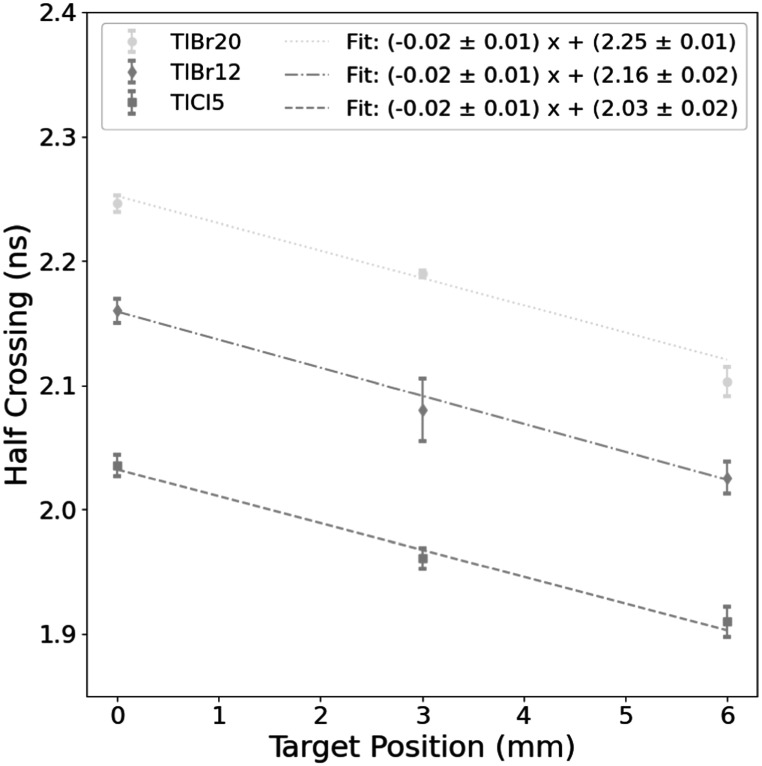
Position versus half crossing of rising edge of TOF distributions for each of the three crystals. Fits and uncertainties are given for each.

The normalized proton time of flight distributions taken as the difference of the gamma-ray timestamp and the T_0_ reference modulo the beam period for each of the three crystals are shown in figure 6. A progressive shift for the 0, 3, and 6 mm positions could be observed for the three crystals. Linear regression fits were applied to the 25–75 percent range of the rising edge of the distributions. The 50 percent crossing of these fits are plotted against the position for each detector with linear fits in figure 7. The *R* ^2^ of all fits was >0.98. The errors for the 50 percent crossings were taken as the error from the fit for the 25, 50, and 75 percent crossing points for all time of flight (TOF) distributions.

**Figure 8.**
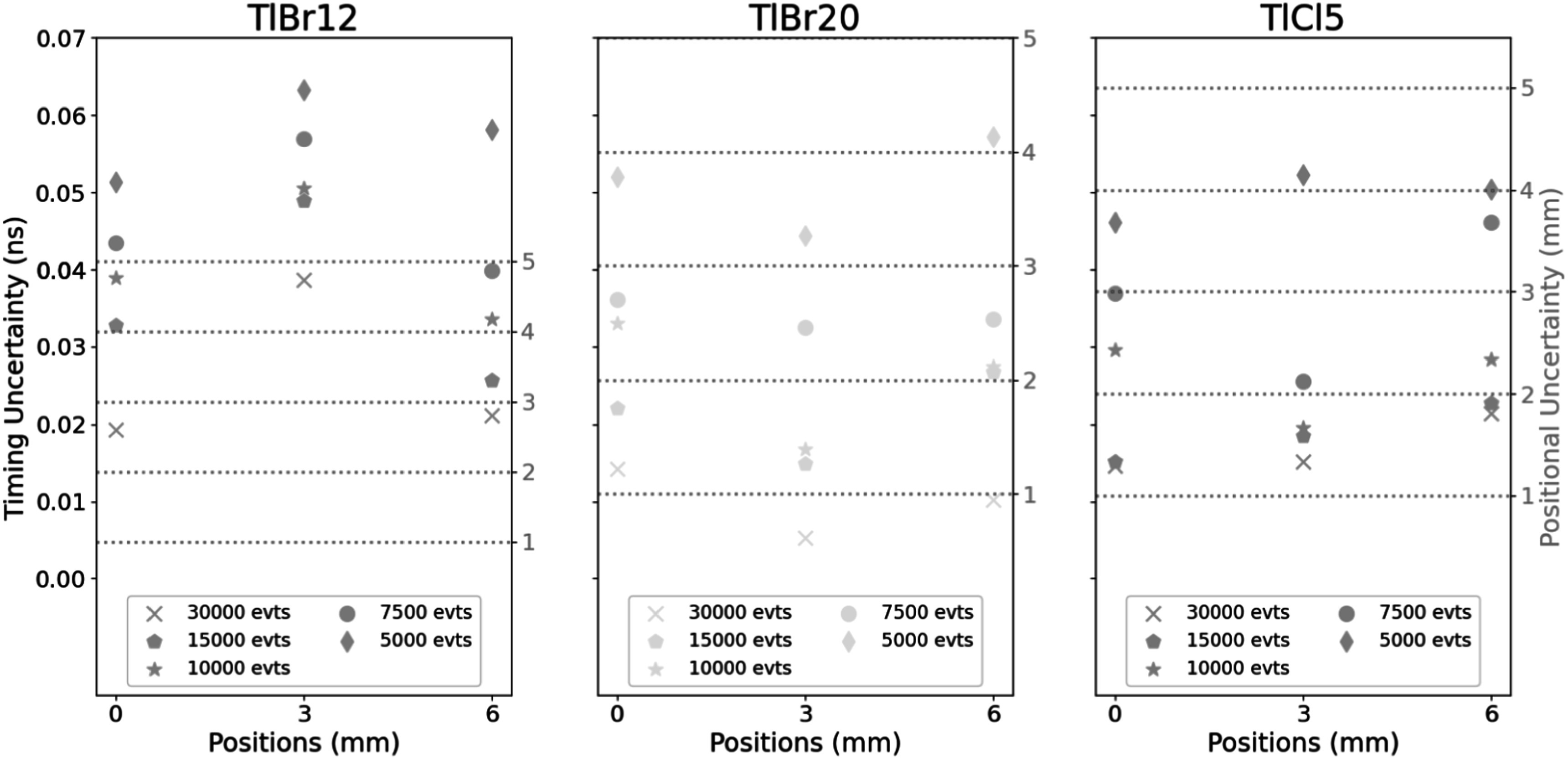
Position versus error for the TlBr12, TlBr20, and TlCl5 crystals with 30k, 15k, 10k, 7.5k, and 5k events from the original acquisition. Also indicated at the right of the figure are the thresholds required for positional accuracy as determined from the fits in figure 7.

Of the three samples, the TlCl5 showed the highest average signal intensity and greatest SNR. To estimate the required number of detected gamma events necessary to achieve a certain position resolution the previous analysis was repeated by random downsampling of the events by factors of 2, 3, 4, and 6 for total events per location of roughly 15 000, 10 000, 7500, and 5000, respectively. The results are presented in figure 8. The uncertainty was given for each point for the crystal at these event levels. The slope of the fit from figure 7 was used to then estimate the required uncertainty to separate shifts of 2, 3, 4, and 5 mm. The initial 1–2 mm accuracy for 30k events was reduced to 4–5 mm when the dataset was downsampled by a factor of 6 (5k events) for TlCl5, which showed the highest accuracy.

## 4. Discussion

The uncorrected 2D distributions in figures 4(a)–(c) show a very strong correlation with the bunch repetition rate of the cyclotron. No neutron-induced continuum (Hueso-González *et al* 2018, Werner *et al* 2019) was present in these distributions. However, we acknowledge that the lower energy of protons at CNL compared to the clinical ones lead to a lower contributions of neutron-induced background. There is no presence either of the 2.2 MeV gamma line from the dexecitation of deuterium following a neutron capture on hydrogen. The lack of solid statistical correlation between the light detected and the energy deposited does not allow for a direct comparison of the energy lines of prompt gammas.

A previous study looking at 511 keV photons in TOF-PET using TlBr and TlCl detectors with very similar crystal dimensions, SiPMs, electronics, and operation conditions, found that this threshold corresponded to at least 5 Cherenkov photons (Ariño-Estrada *et al* 2021a). The same publication reported an average of 3.1 detected Cherenkov photons for 511 keV gamma depositions in TlCl, therefore, it is fair to assume that most events with 5 Cherenkov photons are produced by energy depositions close to 1 MeV of energy.

The current study is the first one, to our knowledge, where the intensity of the Cherenkov light is measured against the detection time to study experimentally the sources of background in PGI measurements done with pure Cherenkov emitters, as shown in figures 4(a)–(c). The differences in crystal dimensions and material choice allowed to extract some preliminary conclusions. Events in the TlCl5 distribution seemed to be more homogeneously distributed for different amplitudes than for the other two cases. This fact could be attributable to the significantly greater Cherenkov yield in TlCl than in TlBr, of approximately 1.5 times for the greatest prompt gamma energies, as recently studied in Rebolo *et al* (2024). The four bars show a saturation feature at 0.5 V (imposed by the DRS4 test board), thus hinting that many events with greater intensity were recorded for this detector that we were unable to resolve in this particular measurement. The cubic shape and full face coverage of this crystal (5 × 5 × 5 mm^3^) do likely optimize the light extraction, widening even more the range of light intensity for this detector compared to the other two.

The TlBr20 dataset showed a greater light intensity than the TlBr12. The match between SiPM and TlBr crystal in the extraction face (3 mm ×3 mm) seems to favor signal intensity better than the total crystal volume. Additionally, the bands at the base of the TlBr12 are wider than in the other two cases. Quantitatively, the SNR values for the TlBr12 and TlCl5 are close with the TlBr20 as an outlier. A possible explanation is that the light collection efficiency is greater because of the larger aspect ratio and one-to-one coupling of SiPM to detector face. Thus, the threshold might have been low enough to include more background. A further study is required to confirm this hypothesis. The SNR is best for the TlCl5 sample despite having smaller SiPM coverage than the TlBr20 and less active volume than the TlBr12. This seems largely a result of the significantly higher signal intensity.

This study is also the first to publish PGT measurements with Cherenkov emitters with beam currents that yield hundreds of protons per bunch. Despite the differences in material properties, dimensions, and fractional photodetector area coverage, the time of flight distributions showed consistent shifts corresponding to the PMMA 3 mm increments towards the beam port. The resulting slopes generated from lines fitted to the rising edge of these distributions is likewise similar between samples within the level of precision of the measurement. The expected velocity of a proton at 67.5 MeV corresponds to a slope of 0.0092 ns mm^−1^, which is within the fitted range.

The accuracy of the PGT method used in these measurements was very sensitive to the number of events collected per dataset. The experimental setup was designed to prove the feasibility of using Cherenkov light in TlBr and TlCl for PGT, while detector dead time and detection efficiency were suboptimal. The disparity in proton bunch width, periodicity, and intensity between clinical proton beams and CNL make it very challenging to predict the behavior of a system based on this type of detector in clinical conditions. It is reasonable to expect a high count rate with this kind of detector coupled to fast readout electronics and thus be able to acquire events quickly in beams with very high intensity. Validation of these expectations, which would require acquiring data at beams with very high currents, are beyond the scope of this work.

## 5. Conclusion

A PGT acquisition setup consisting of Cherenkov detectors was operated in a proton beam line with currents comparable to those used in the clinical proton therapy treatments. Time of flight distributions were generated with a moving target with a T_0_ and TlBr and TlCl prompt gamma detectors. The SNR was found to be high across all three samples and the shifts observed were indicative of the expected proton time of flight. This is additionally promising for the TlBr and TlCl crystals since, as semiconductors, a separate and independent charge induction signal could be used for fine energy spectroscopy and finer event selection.

The results presented here might be further improved through the use of a T_0_ detector with more consistent signals between pulses and less saturation effects. These results encourage the use of Cherenkov signals for PGT, either as a stand-alone signal or simultaneously with a parallel readout that provides complementary information.

## Data Availability

The data cannot be made publicly available upon publication because they are not available in a format that is sufficiently accessible or reusable by other researchers. The data that support the findings of this study are available upon reasonable request from the authors.
